# Urban Versus Rural Disparities in Alcoholic Liver Disease Mortality: A Comprehensive 22-Year Analysis of Trends and Demographic Influences in the United States

**DOI:** 10.7759/cureus.86362

**Published:** 2025-06-19

**Authors:** Kesha Pathak, Najeeha A Bhatti, Yashaswi Guntupalli, Bharat Vejandla, Kanishka Srinivasan, Kinjal Shah

**Affiliations:** 1 Internal Medicine, Gujarat Adani Institute of Medical Sciences (GAIMS), Bhuj, IND; 2 Medicine, Rawalpindi Medical University, Rawalpindi, PAK; 3 Internal Medicine, Sri Venkateswara Institute of Medical Sciences (SVIMS), Tirupati, IND; 4 Internal Medicine, All American Institute of Medical Science, Black River, JAM; 5 Internal Medicine, Sree Balaji Medical College and Hospital, Chennai, IND; 6 Health Administration, Edward J. Bloustein School of Planning and Public Policy, New Brunswick, USA

**Keywords:** alcoholic liver disease, epidemiology, health outcomes, mortality, socioeconomic, urban-rural disparities

## Abstract

Introduction

Alcoholic liver disease (ALD) encompasses a spectrum of liver conditions that result from excessive alcohol consumption. Disparities in mortality rates of ALD between urban and rural populations have become a growing concern, with rural areas experiencing a faster rise in ALD-related deaths. Understanding these disparities is crucial for developing targeted public health interventions to address this escalating concern.

Methodology

Disparities in mortality rates from 1999 to 2020 were analyzed using data from the CDC-WONDER (Centers for Disease Control and Prevention, Wide-ranging Online Data for Epidemiologic Research) database. Regions were categorized into urban (large central, large fringe, medium, and small metro) and rural (micropolitan and non-core) categories based on the 2013 Metropolitan classification. The data were stratified by age (10-year intervals), gender, and race. The binomial proportion test was used for comparison, and a p-value < 0.05 was considered significant.

Results

Rural mortality rates increased sharply post-2015, peaking in 2020, while urban rates rose more gradually. While urban areas experienced higher total mortality, rural regions exhibited a steeper rise in mortality rates, especially post-2015, highlighting a growing public health crisis. Notably, younger age groups (15-64 years) exhibited higher mortality in rural areas, particularly among those aged 25-34 years, while urban areas showed higher rates for older populations (65+ years). American Indian or Alaska Native individuals in rural areas had notably high rates (24.1 per 100,000). Rural males experienced higher mortality rates (9.04 per 100,000) compared to urban males (7.82 per 100,000, p < 0.001). Similarly, rural females also exhibited higher mortality (3.48 per 100,000) compared to urban females (3.08 per 100,000, p < 0.001), highlighting a consistent rural disadvantage across both genders.

Conclusions

The findings of this study indicate that males and middle-aged adults in rural settings are disproportionately affected. Targeted public health interventions aimed at improving healthcare access can significantly reduce ALD mortality rates.

## Introduction

Alcoholic liver disease (ALD) is not just a medical concern; it is a social and systemic crisis affecting millions globally. It comprises a spectrum of conditions that includes alcoholic fatty liver, which may present with or without hepatitis. Acute ingestion of alcohol can result in alcoholic hepatitis, a condition that is often reversible. In contrast, cirrhosis denotes the irreversible stage of the disease. Individuals with severe alcohol use disorder are particularly vulnerable. They have a heightened likelihood of developing chronic liver disease (CLD). CLD is a significant consequence of excessive alcohol consumption. Understanding this spectrum is essential for effective clinical management.

Alcohol-associated liver disease constitutes 5.1% of global disease burden, ranking 30th in causes of death [1]. In the U.S., alcohol use results in approximately 178,000 deaths annually, with individuals aged 50-64 facing the highest mortality [2,3]. Understanding the disparities in mortality rates between urban and rural populations is crucial for developing effective prevention and treatment strategies. The limited research addressing these disparities has necessitated the undertaking of this study. In 2018, the variation in alcohol-related mortality rates between urban and rural areas was more substantial than in 2000 for both males and females aged 25 and older [4]. Understanding urban-rural disparities is critical for informing future public health strategies.

This understanding is key to developing future strategies. It informs the design of targeted public health interventions. By addressing these disparities, we can improve health outcomes. Effective prevention strategies are crucial for ongoing challenges. Focusing on these areas can lead to more equitable solutions. The lack of research examining the disparities in mortality rates between urban and rural populations for alcohol liver disease prompted us to undertake this study.

## Materials and methods

Aims and objectives

Our study aims to evaluate disparities in mortality rates between urban and rural regions for ALD within the United States, using the CDC-WONDER database.

Methodology

A retrospective original research study was conducted using the CDC-WONDER (Centers for Disease Control and Prevention, Wide-ranging Online Data for Epidemiologic Research) database to determine disparities in mortality rates between urban and rural regions for ALD within the United States [5]. The data used in this study were extracted on August 20, 2024. Since the data from CDC-WONDER is de-identified, publicly available, and no human participants are involved, ethics committee approval was not required for this study. While the dataset is de-identified and ethics approval was not required, this study acknowledges the potential limitations of utilizing secondary data.

The data collection was conducted on a single day (August 20, 2024), using the CDC-WONDER database. The option "Underlying causes of death" was used for the query on the website, and "1999-2020: Underlying Cause of Death by Bridged-Race Categories" and the ICD-10 code K-70 (Alcoholic Liver Disease) were selected. Subsequently, the selection was made for the 2013 Metropolitan classification, which was categorized into two groups: (i) urban/metropolitan, comprising categories such as large central, large fringe, medium, and small metro, and (ii) rural/non-metropolitan, including micropolitan and non-core. These data are grouped by variables such as age (in 10-year intervals), gender, and race. These variables were selected to capture demographic and socioeconomic disparities that impact ALD mortality trends.

Data were then exported to a Microsoft Excel sheet (Microsoft Corporation, Redmond, Washington) for further analysis. Statistical analysis was done using the R core team (2023) R: A Language and Environment for Statistical Computing (The R Foundation for Statistical Computing, Vienna, Austria). The plot was created using GGPlot 2: Elegant Graphics for Data Analysis (Springer-Verlag New York, 2016).

## Results

Aggregate data of 373,302 deaths from 1999 to 2020 due to ALD were obtained from the CDC-WONDER database. The data extraction included variables such as age, sex, race/ethnicity, geographic region, and year of death. This comprehensive dataset allowed for an in-depth analysis of mortality trends over the two-decade period, facilitating a better understanding of the impact of ALD across different demographics and regions in the United States.

Table 1 shows the absolute number of reported mortalities in urban and rural areas due to ALD from 1999 to 2020 as per the 2013 Urbanization Classification. The total number of deaths in rural areas was 62,982, and in urban areas, it was 310,320, from 1999 to 2020.

**Table 1:** Absolute number of reported mortalities from alcoholic liver disease. Urban areas: large central metropolitan, large fringe metropolitan, medium metropolitan, small metropolitan Rural areas: micropolitan, non-core (non-metropolitan)

Type	n (%)
Urban (metropolitan area)	310,320 (83.1%)
Large central metropolitan	117,567 (37.8%)
Large fringe metropolitan	70,193 (22.6%)
Medium metropolitan	84,581 (27.25%)
Small metropolitan	37,979 (12.2%)
Rural (non-metropolitan area)	62,982 (16.9%)
Micropolitan	37,045 (58.9%)
Non-core	25,937 (41.1%)

**Figure 1:**
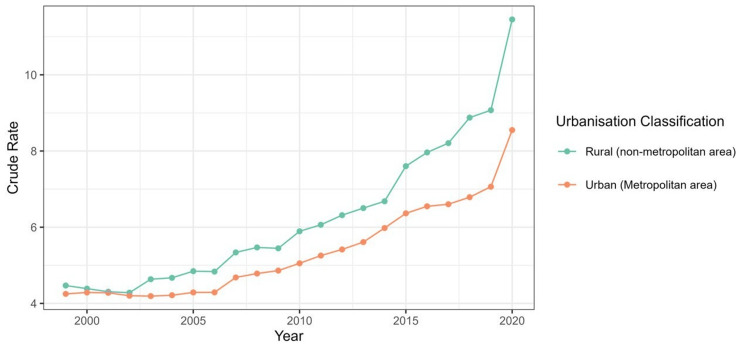
Line diagram illustrating the widening urban-rural gap in crude mortality rates, with rural areas experiencing a sharper rise post-2015.

Figure 1 displays a line diagram that shows trends in urban versus rural mortality due to ALD, calculated as a crude rate per 100,000 population.

Rural vs. urban trends

In Figure 1, the green line represents rural (non-metropolitan) areas, while the orange line represents urban (metropolitan) areas. Rural areas show a steady increase in the crude rate, with a sharp rise from around 2017 onwards, reaching its peak in 2020. Urban areas show that the crude rate also rises, but at a slower pace compared to rural areas. Urban areas show a more gradual increase, with a significant jump starting around 2019.

Comparative analysis

From 1999 to 2015, both urban and rural areas maintained relatively low and stable rates. Rural areas consistently show a higher crude rate than urban areas during this time. Post-2015, there has been a more noticeable divergence between rural and urban rates. Rural areas experience a sharper increase, especially from 2017 onward, leading to a larger gap between the two by 2020. Both rural and urban areas see a significant rise in the crude rate by 2020, but the increase is far more pronounced in rural areas.

Figure 1 also indicates that while both rural and urban areas have experienced an increasing trend in crude rates over the two decades, rural areas have seen a much steeper rise in recent years. This suggests that rural areas may be disproportionately affected by the underlying cause compared to urban areas, particularly in the final years of the data. The pronounced increase in rural mortality during 2020 may reflect socioeconomic disruptions exacerbated by the COVID-19 pandemic.

Table 2 provides a breakdown of mortality due to ALD based on age, gender, and race across urban and rural populations, with the corresponding binomial test p-values for statistical significance (*p-value less than 0.005). All differences in mortality rates, except for the White population, were statistically significant, highlighting distinct urban-rural disparities.

**Table 2:** Mortality due to alcoholic liver disease in urban and rural areas based on age, gender, and race, starting from 15 years of age up to 85+ years of age. Ages 0-14 were not selected in this study. * P-value less than 0.005

Variables	Urban			Rural			Binomial Test
	Mortality	Total Population	Mortality Rate (per 100,000)	Mortality	Total Population	Mortality Rate (per 100,000)	p-value
Age groups							
15-24 years	291	799,755,615	0.04	71	136,038,105	0.05	0.003*
25-34 years	8832	803,054,375	1.1	1792	117,033,859	1.53	<0.001*
35-44 years	38,359	803,916,304	4.77	7619	127,369,780	5.98	<0.001*
45-54 years	94,527	788,609,639	11.99	18,595	138,965,498	13.38	<0.001*
55-64 years	100,495	639,136,856	15.72	21,105	127,286,485	16.58	<0.001*
65-74 years	48,841	417,988,177	11.68	10,192	92,469,123	11.02	<0.001*
75-84 years	16,204	244,183,949	6.64	3170	54,320,144	5.84	<0.001*
85+ years	2736	98,324,522	2.78	433	21,189,280	2.04	<0.001*
Gender							
Male	220,175	2,815,055,490	7.82	45,426	502,292,400	9.04	<0.001*
Female	90,145	2,924,420,159	3.08	17,556	504,579,252	3.48	<0.001*
Race							
American Indian or Alaska Native	7158	62,750,775	11.41	6171	25,610,416	24.1	<0.001*
Asian or Pacific Islander	4473	359,973,366	1.24	198	11,940,089	1.66	<0.001*
Black or African American	27,817	830,766,851	3.35	3366	88,267,994	3.81	<0.001*
White	270,872	4,485,984,657	6.04	53,247	881,053,153	6.04	0.837

Age groups

In the 15-24-year interval, mortality rates are slightly higher in rural areas (0.05 per 100,000) than urban areas (0.04 per 100,000), with a statistically significant p-value of 0.003. In the interval of 25-34 years, the mortality rate in rural areas (1.53 per 100,000) is significantly higher than in urban areas (1.1 per 100,000), with a p-value of <0.001, indicating a strong disparity. Rural areas again show higher mortality (5.98 per 100,000) compared to urban areas (4.77 per 100,000) in the age group of 35-44 years. The difference is statistically significant (p < 0.001). In the age group of 45-54 years, rural areas exhibit a higher mortality rate (13.38 per 100,000) than urban areas (11.99 per 100,000), with a significant p-value of <0.001, similar to the interval of 55-64 years, where mortality in rural areas (16.58 per 100,000) is slightly higher than in urban areas (15.72 per 100,000), with statistical significance (p < 0.001).

In contrast, the 65-74 age group exhibits a higher mortality rate in urban areas (11.68 per 100,000) compared to rural areas (11.02 per 100,000), although the difference remains statistically significant (p < 0.001). In the age group 75-84 years, mortality is lower in rural areas (5.84 per 100,000) compared to urban areas (6.64 per 100,000), which is statistically significant (p < 0.001). Lastly, the mortality rate for individuals aged 85 and above is lower in rural areas (2.04 per 100,000) compared to urban areas (2.78 per 100,000), with statistical significance (p < 0.001).

Mortality due to ALD is generally higher in rural areas for age groups 15-64 years, with the trend reversing in older age groups (65+), where urban areas show higher mortality. Both males and females in rural areas exhibit higher mortality rates compared to their urban counterparts, with males being more affected overall. American Indian or Alaska Native individuals in rural areas are particularly vulnerable, showing a much higher mortality rate. All racial groups except Whites show higher mortality in rural areas. There is a general trend of higher mortality rates due to ALD in rural areas, especially among younger populations, males, and racial minorities, particularly American Indian or Alaska Native individuals. Urban areas show higher mortality rates in older age groups (65+), particularly among those 75+. Most differences between rural and urban areas are statistically significant, except for the White population, where the mortality rates are identical. These findings suggest that rural areas may require more targeted interventions, particularly for younger adults, men, and specific racial groups, to address the higher burden of ALD mortality.

## Discussion

The study investigates the mortality rates due to ALD across urban and rural areas. Urban areas report more total mortalities (310,320) than rural areas (62,982). However, when accounting for population size, rural areas exhibit a steeper rise in mortality rates, particularly after 2015, indicating a more rapidly growing crisis. Urban areas consistently experienced higher mortality rates than rural areas throughout the study period. The gap between urban and rural mortality rates has widened over time, particularly for the 25-44 age group. Metropolitan areas had the highest mortality rates, followed by large central metropolitan, large fringe metropolitan, medium metropolitan, small metropolitan, and rural areas. Rural areas with a micropolitan population showed higher mortality rates than those without. Middle-aged adults (25-54 years) were most affected, with significantly higher mortality rates in both urban and rural areas compared to other age groups. The risk of alcohol-related liver disease increases with age, reaching its peak in middle adulthood. While mortality rates remain high in the older age groups, there is a slight decrease in the 75+ age group. Males consistently had higher mortality rates than females. Among racial groups, American Indians or Alaska Natives had the highest mortality rates, followed by Black or African Americans. Whites had lower mortality rates compared to other racial groups, with no significant difference between urban and rural areas. All variables except the White population exhibit a statistically significant difference in mortality rates between urban and rural settings.

Studying urban and rural mortality is crucial because these areas often face distinct socioeconomic, healthcare access, and lifestyle challenges that influence health outcomes differently. Understanding these differences enables the tailoring of public health interventions to address the specific needs of each area. This data indicates a higher crude mortality rate in urban areas compared to rural ones, as shown in both the table and the line graph. Urban environments may foster lifestyles that encourage higher alcohol consumption, such as increased stress, alcohol availability, and social environments conducive to drinking. Urban diets often include more processed foods, which can contribute to liver stress and associated diseases. Despite proximity, some urban populations might experience barriers to healthcare access due to costs or overburdened systems. Higher stress levels associated with fast-paced urban living, along with disparities in income, may lead to both increased alcohol use and limited preventive healthcare. Urban areas generally have better access to healthcare services, which may explain the lower mortality rate despite the higher absolute number of deaths. Rural areas might experience delayed diagnoses or less frequent access to medical care, contributing to the rapid rise in mortality rates. Rural areas often have higher poverty levels, which can lead to poor health outcomes, including increased alcohol consumption and limited access to preventive healthcare, compounding the issue of liver disease. Higher alcohol use may be prevalent in certain rural populations due to cultural or social factors, leading to a disproportionate impact of ALD in these areas, as seen in the significant increase in crude mortality rates over time.

Disparities in mortality by race, particularly the higher rates among American Indian or Alaska Native populations, may point to genetic predispositions, higher alcohol consumption, or socioeconomic stressors that are more pronounced in rural areas. Rural areas may lack adequate healthcare infrastructure, leading to delayed diagnosis and treatment of alcohol-related liver disease. This could indicate the need for improved healthcare access, including more specialized services in these regions. Higher rural mortality may reflect greater socioeconomic challenges, such as poverty, unemployment, and limited educational opportunities, which can lead to increased alcohol consumption and poorer overall health. It may signify that rural populations have higher alcohol consumption or less engagement in preventive health behaviors compared to urban populations, leading to more advanced stages of liver disease when diagnosed.

Social isolation, cultural norms around alcohol use, or occupational stress (such as in agriculture or manual labor) could be driving factors for higher mortality in rural regions, necessitating targeted lifestyle interventions. Rural disparities are driven by various structural factors, especially in areas with high minority populations. For instance, rural Black residents, primarily in the southern U.S., face greater challenges due to the lack of Medicaid expansion in many states [6]. Additionally, rural minority populations often experience higher poverty rates and lower educational attainment, contributing to increased mortality risks and limited resources in these communities [6]. ESLD (end-stage liver disease) patients in rural hospitals had higher odds of in-hospital death than those in urban hospitals. This was not due to patient factors. It suggests that improving care for these patients requires focusing on hospital-level interventions [7]. Rural residents experience worse health outcomes than urban residents, including higher rates of chronic diseases, mental health issues, and disabilities. These disparities have widened over time due to inequalities in socioeconomic factors, access to healthcare, and environmental conditions [8]. Rural children also face unique health challenges, including higher rates of obesity and exposure to unhealthy environments. Addressing these underlying social determinants of health is crucial for reducing rural-urban health inequalities [8].

This study is consistent with a similar study by Esser et al. [9]. Both studies showed an alcohol-related mortality, highlighting increases in deaths attributed to alcohol use. The study highlights higher mortality rates among males and also notes that although more males died from excessive alcohol use, the rate of increase in deaths was higher among females. This study is consistent with a similar study in identifying a recent worsening in the situation [9]. The study by Esser et al. further points to a steep rise in rural areas post-2015; the other study discusses a significant increase in alcohol-related deaths during 2020-2021, partly attributed to the COVID-19 pandemic and increasing binge drinking in those aged 35-50 [9]. This study spans 22 years, focusing on changes over a longer period, particularly after 2015, while specifically examining the five-year period from 2016 to 2021, with detailed attention to the pandemic years (2020-2021). Esser et al. explicitly discussed the role of the COVID-19 pandemic in increasing alcohol-related deaths, pointing to factors like expanded alcohol access and mental health challenges. This aspect is absent from this manuscript, which focuses on longer-term trends and does not mention the pandemic. This study highlights the disproportionate impact of ALD on American Indian or Alaska Native populations, which is also proven in another study by Kulkarni et al. [10] that emphasizes this group's higher mortality rates despite lower overall alcohol consumption, pointing to systemic failures like inadequate healthcare as contributing factors and acknowledges ALD as a growing crisis [10].

This study is consistent with Kulkarni et al. and acknowledges that men are more affected by ALD than women. The finding of this study also shows similarities with the findings of another study by Ilyas et al. [11], which highlights significant increases in ALD-related mortality rates. Ilyas et al. reported a more pronounced increase in ALD-related mortality among females, which opposes the findings of this study, emphasizing that males have significantly higher mortality overall. This study aligns with Ayyala et al. [12] and underscores that, although urban areas exhibit higher total mortality rates, rural areas are experiencing a faster-growing mortality crisis, particularly after 2015.

Rural areas, when accounting for population size, are described as having a steeper rise in ALD mortality rates. Ayyala et al. focused more on the trends in age-adjusted mortality rates (AAMR) and provided detailed annual percentage changes (APC) over time, emphasizing the period between 1999 and 2019 and how APC has evolved in different populations, and also pointed out that women in rural areas have seen a more significant increase in ALD mortality (+5.7% vs. +4.6% for men), thus opposing the findings of this study. This study presents broader results, summarizing total mortality numbers for urban and rural areas without discussing specific APC or regional breakdowns. While this study notes that mortality is generally higher among males, it does not further investigate the details of APC trends by gender.

A different study by Wang et al. [13] reported a decrease in age-standardized mortality rates for Black individuals and an increase for White individuals, which contradicts the findings of this study, highlighting different factors like alcohol-related liver disease and metabolic dysfunction in Whites and viral hepatitis tapering in Blacks. The mortality trends were more pronounced among younger populations (ages 25-64) than among older populations (65+), according to Wang et al. This study, however, shows that mortality is highest in middle adulthood (ages 35-54), with a slight decline in the older population (75+).

ALD mortality was noted to be highest among middle-aged Native Americans and rural residents, with significant increases across most groups since 2006, except for non-Hispanic Black men. Native American women saw the largest rise (2005-2017 ARD 0.8), followed by non-Hispanic White men (2006-2017 ARD 0.4) and women (2013-2017 ARD 0.4), as per the study by Moon et al. [14]. A similar cohort study using U.S. vital statistics data from 1999 to 2020 analyzed alcohol-induced deaths. The findings revealed a peak in deaths among individuals aged 45 to 60 [15]. It was observed that there was a significant increase in alcohol-induced deaths across all age groups, particularly after 2010. This trend suggests a growing public health concern related to alcohol consumption [15].

People with disabilities, unemployed people, and those not in the labor force were more likely to die from ALD than those employed, as per Kposowa et al. [16]. These factors were strongly related to ALD mortality regardless of gender, race, and ethnicity. Other factors such as being male, minority status, urban living, renting, lower education, marital status, low income, and age were also associated with ALD mortality [16]. Another study found that Black individuals, older patients, those with multiple chronic conditions, and patients in urban teaching hospitals or large hospitals had higher mortality rates [17]. Private insurance and lack of insurance were also associated with increased mortality. In contrast, Hispanic race, higher income, and mid-west hospitals were linked to lower mortality. These findings highlight the importance of addressing racial disparities, improving care for older patients and those with comorbidities, and ensuring equitable access to healthcare [17].

Given the retrospective, descriptive, and ecological nature of this study, no causal inferences can be drawn. The future scope of work in this field involves investigating the specific causes behind disparities in urban and rural mortality rates related to ALD. Researchers need to explore whether these differences are driven by socioeconomic factors, such as poverty or lack of education, or by unequal access to healthcare facilities. Delays in transportation in rural areas may also contribute to worse outcomes, making timely treatment more challenging. Additionally, the availability of advanced healthcare services in urban settings versus rural areas could affect survival rates. Complications arising from the disease itself may also play a role, as rural patients may have limited access to specialized treatment. By identifying these factors, healthcare policies can be better tailored to reduce the disparity between urban and rural populations. Rural public health initiatives should prioritize improving access to healthcare, addressing socioeconomic stressors, and reducing barriers to preventive care.

Limitations

This study does not include data from the years 2021 to 2023, limiting the analysis to trends and mortality rates up to 2020. As a result, recent shifts in mortality due to ALD during this period are not captured. The study did not classify ALD into specific sub-categories, which may limit the understanding of different forms or stages of the disease and their respective impacts on mortality rates. Specific causes of death directly related to ALD were not analyzed in this study. The data used was derived from the CDC-WONDER database, which does not provide detailed listings of causes directly associated with ALD. The CDC-WONDER data does not provide sufficient information on these variables, which limits a more comprehensive understanding of external influences on mortality rates in urban versus rural populations. This acknowledges the limitations regarding timeframes, disease categorization, and the absence of socioeconomic and healthcare access data in this analysis of ALD mortality. The study is limited by the lack of post-2020 data, which prevents an analysis of recent trends, including the long-term impacts of the COVID-19 pandemic on ALD mortality.

## Conclusions

The evaluation of ALD mortality rates in the United States over the 22-year period showed significant disparities between urban and rural regions. The findings showed that rural regions showed a higher rate of ALD-related mortality compared to urban regions. Furthermore, the study found that males and older patients were significantly affected by ALD mortality. This shows that further research is needed to dive deeper into the underlying causes leading to persistent rural disparities, particularly among rural men. This research highlights the need for tailored public health strategies to address urban-rural disparities in ALD mortality, focusing on improving healthcare access and tackling socioeconomic challenges. By addressing these knowledge gaps, policymakers and healthcare providers can develop targeted strategies to improve the health outcomes of individuals with ALD, particularly in rural areas. These strategies could include access to preventive care, addressing socioeconomic factors that contribute to alcohol abuse and improving treatment options. Addressing these effective interventions ultimately reduces the overall burden of this disease.
